# The human milk microbiome aligns with lactation stage and not birth mode

**DOI:** 10.1038/s41598-022-09009-y

**Published:** 2022-04-04

**Authors:** Katriona E. Lyons, Carol-Anne O.’ Shea, Ghjuvan Grimaud, C. Anthony Ryan, Eugene Dempsey, Alan L. Kelly, R. Paul Ross, Catherine Stanton

**Affiliations:** 1 Teagasc Food Research Centre, Moorepark, Fermoy, Cork Ireland; 2 APC Microbiome Ireland, University College Cork, Cork, Ireland; 3 School of Microbiology, University College Cork, Cork, Ireland; 4 Department of Neonatology, Cork University Maternity Hospital, Cork, Ireland; 5 School of Food and Nutritional Sciences, University College Cork, Cork, Ireland

**Keywords:** Cell biology, Computational biology and bioinformatics, Microbiology, Molecular biology

## Abstract

We analysed the human milk microbiome in a cohort of 80 lactating women and followed the dynamics in taxa over the course of lactation from birth to 6 months. Two hundred and thirty one milk samples were collected from full-term lactating women at 1, 4, 8 and 24 weeks following birth and analysed for microbiota composition using 16S rRNA sequencing. A significant decrease in milk microbiota diversity was observed throughout the first 6 months of lactation, with the greatest difference seen between week 8 and week 24. Nine genera predominated in milk over lactation from week 1 to week 24, comprising of *Staphylococcus*, *Streptococcus*, *Pseudomonas*, *Acinetobacter, Bifidobacterium, Mesorhizobium, Brevundimonas, Flavobacterium,* and *Rhodococcus*; however, fluctuations in these core genera were apparent over time. There was a significant effect of stage of lactation on the microbiome, while no effect of birth mode, infant sex and maternal BMI was observed throughout lactation. *Streptococcus* had the highest mean relative abundance at week 1 and 24 (17.3% and 24% respectively), whereas *Pseudomonas* predominated at week 4 (22%) and week 8 (19%). *Bifidobacterium* and *Lactobacillus* had the highest mean relative abundance at week 4 (5% and 1.4% respectively), and occurred at a relative abundance of ≤ 1% at all other time points. A decrease in milk microbiota diversity throughout lactation was also observed. This study concluded that lactation stage was the primary driving factor in milk microbiota compositional changes over lactation from birth to 6 months, while mode of delivery was not a factor driving compositional changes throughout human lactation.

## Introduction

Human breast milk is the gold standard feeding regime for new-born infants, and has long been recognised for its benefits for enhancing infant health, growth, and development. The World Health Organization (WHO) and The United Nations Children’s Fund (UNICEF) recommend that breastfeeding should be initiated within the first hour after birth and that infants should be exclusively breastfed for the first 6 months^1^.

Breast milk contains the optimal amount of macro and micronutrients, and provides complete nutrition for the infant^2^. In addition to these nutrients, breast milk contains an array of bioactive and immune factors such as antibodies, lysozyme, growth factors, antimicrobial peptides, microRNAs, stem cells and human milk oligosaccharides (HMOs) which all most likely influence the developing infant immune system and provide defence against pathogens^3–5^. Furthermore, breast milk is a source of beneficial bacteria such as *Bifidobacterium* and *Lactobacillus,* which are known for their health promoting benefits, and HMOs can support the proliferation of these potential probiotic microorganisms in the infant gut^6–8^. As such, the intake of breast milk throughout lactation assists in the formation of the infant gut microbiota and consequently aids in the development and maturation of the new-born immune system^9^.

The microbiome of human milk in early lactation has been investigated in a number of short term studies to date, with over several hundred bacterial species identified^10–12^. While these investigations determined the milk microbiome of small cohorts of healthy women during early lactation (≤ 1 month), differences in core genera among these studies are evident. Indeed, it is important to note that breast milk composition varies among individuals, and it is contingent on many factors, such as maternal diet, genetics, health, antibiotic usage, mode of delivery, demographic, and environmental differences^13–18^.

Few studies have documented the evolution and progression of bacterial communities in human milk over time. Murphy et al. (2017) evaluated the bacterial species in milk and infant stool in ten mother-infant pairs from birth to three months. The milk microbiota consisted of 12 core genera: *Pseudomonas, Staphylococcus, Streptococcus, Elizabethkingia, Variovorax, Bifidobacterium, Flavobacterium, Lactobacillus, Stenotrophomonas, Brevundimonas, Chryseobacterium* and *Enterobacter*^19^. Limited studies have examined the human milk microbiota up to 6 months of lactation. Cabrera-Rubio et al. characterized the milk microbial communities in 18 mothers at 2 days, 1 month and 6 months lactation using pyrosequencing and qPCR. Distinct compositional changes were detected between colostrum and 6 month milk samples in this study, while higher maternal body mass index (BMI) was associated with less diverse bacterial communities in milk^20^. Gonzales et al. investigated the milk microbiota at two time points from different participants at 6–46 days post-partum and 109–184 days post-partum; a shift in microbiota composition was observed, with *Staphylococcus* and *Streptococcus* being present in higher abundances in early-lactation milk and *Sphingobium* and *Pseudomonas* more abundant in later milk^21^. However, many of these investigations are constrained by factors such as sample size, lack of pairwise longitudinal sampling and dated scientific approaches such as qPCR and pyrosequencing^20,21^.

In this study, we investigated the evolution of milk microbiome composition throughout human lactation to 6 months in 80 lactating women, analyzing two hundred and thirty one breast milk samples using 16S rRNA compositional sequencing, while also investigating the impact of perinatal factors on the evolving milk microbiome throughout lactation.

## Materials and methods

### Subjects and sample collection

This study was approved by the Clinical Research Ethics Committee of the Cork Teaching Hospitals. All participants enrolled in this study provided written informed consent and all relevant guidelines and regulations were followed. Of the 118 full term, breastfeeding women who enrolled in the INFAMILK study, 82 provided a milk sample at one or more time points (1 week (n = 67), 4 weeks (n = 65), 8 weeks (n = 54) and 24 weeks (n = 42)). Participants were recruited at Cork University Maternity Hospital. Recruits were healthy lactating women who had given birth at full term via vaginal or Caesarean-section (C-section) deliveries. Inclusion criteria were as follows; gestation age ≥ 37 weeks, birth weight > 1500 g. The clinical characteristics of the participants are shown in Supplementary Information Table S1. Milk samples were collected from each mother at 1, 4, 8 and 24 weeks following birth. For milk sampling, mothers were asked to clean the breast with water and express 15–20 mL of milk into a sterile container provided and store at 4 °C until collection and delivery to the laboratory. Milk samples were subject to processing within 24 h of donation.

### DNA extractions

Microbial DNA was extracted from 231 breast milk samples using a modified protocol from the DNeasy Powerfood Microbial kit (Qiagen, UK) (formerly PowerFood™ Microbial DNA Isolation kit, MoBio, Carlsbad, CA) as previously outlined^22^. Briefly, milk samples were subjected to initial centrifugation of 4000*g* × 30 min at 4 °C, the fat layer removed with a sterile cotton swab (Thermo Fisher Scientific, Inc.), and the supernatant was discarded. Cell pellets were washed twice with phosphate buffered saline (Sigma Aldrich) and treated with 90 µL of 50 mg/mL lysozyme (Sigma Aldrich) and 50 µL of 5 KU/mL mutanolysin (Sigma Aldrich) followed by incubation at 55 °C × 15 min. Samples were subsequently treated with 28 µL of 20 mg/mL proteinase k (Qiagen, UK) and incubated further at 55 °C for 15 min followed by using the DNeasy PowerFood Microbial kit protocol. Negative controls using sterile molecular water (Sigma Aldrich) were extracted as above.

### Preparation of DNA for MiSeq sequencing

Using the 16S metagenomic sequencing library protocol (Illumina) the V3-V4 hypervariable region of the 16S rRNA gene was amplified from 231 milk DNA extracts as previously outlined^22^. Two negative controls were included in this study, an extraction kit control and a PCR control. DNA was amplified with specific primers to the V3-V4 region; forward primer 5’ TCGTCGGCAGCGTCAGATGTGTATAAGAGACAGCCTACGGGNGGCWGCAG; and reverse primer 5’ GTCTCGTGGGCTCGGAGATGTGTATAAGAGACAGGACTACHVGGGTATCTAATCC. Each PCR reaction contained template DNA, 1 µL forward primer (5 µM), 1 µL reverse primer (5 µM), 12.5 µL 2X Kapa HiFi Hotstart ready mix (Anachem, Dublin, Ireland) and PCR grade water. PCR conditions for amplification consisted of heated lid 110 °C, initial denaturation at 95 °C × 3 min, 25 cycles of 95 °C × 30 s, 55 °C × 30 s, 72 °C × 30 s, followed by 72 °C × 5 min and holding at 4 °C. PCR products were visualised using gel electrophoresis (1X TAE buffer, 1.5% agarose, 100 V) to confirm amplification. Amplicons were cleaned using AMPure XP magnetic bead based purification (Labplan, Dublin, Ireland) and subject to a second PCR reaction. Illumina sequencing adapters and dual‐index barcodes (Illumina Nextera XT indexing primers, Illumina, Sweden) were added to the purified DNA (5 µL) to index each of the samples, allowing the library to be pooled for sequencing as outlined in the Illumina library preparation protocol. Samples were quantified using the Qubit (Bio-Sciences, Dublin, Ireland), along with the broad range DNA quantification assay kit (BioSciences) and samples were then pooled in an equimolar fashion. The pooled sample was run on the Agilent Bioanalyser for quality analysis prior to sequencing. The sample pool was prepared following Illumina guidelines. Samples were sequenced on the MiSeq sequencing platform in the Teagasc Sequencing facility, using a 2 × 300 cycle V3 kit, following standard Illumina sequencing protocols.

### Bioinformatic and statistical analysis

Three hundred base pair paired-end reads were assembled using FLASH (FLASH: fast length adjustment of short reads to improve genome assemblies)^23^. QIIME was used for further processing of paired-end reads, and quality filtering was based on a quality score of > 25 and removal of mismatched barcodes and sequences below length thresholds^24^. Denoising, chimera detection and clustering into operational taxonomic units (OTUs) were performed in QIIME using USEARCH v7 (64-bit) 3^25^. OTUs were assigned using PyNAST (PyNAST: python nearest alignment space termination; a flexible tool for aligning sequences to a template alignment) and taxonomic rank was assigned using BLAST against the SILVA SSURef database release v123^26,27^. Samples with < 10,000 reads were excluded.

Statistical analysis was performed using Calypso online software (version 8.84). Relative abundances were calculated in Microsoft Excel and R (version 4.02)^28^ using the R packages phyloseq v1.34.0^29^, microbiomeutilities v0.99.02^30^ and ggplot2 v3.3.3^31^. In Calypso, cumulative-sum scaling was used to normalise microbial community data and data were log2 transformed to account for the non-normal distribution of taxonomic count data for alpha and beta diversity testing. Alpha diversity was determined using the Shannon, Simpson and Chao1 indices. Beta diversity was measured using Principal Coordinate Analysis (PCoA) based on Bray–Curtis distance matrices on data. Multivariate analysis was examined using Redundancy analysis (RDA) and Canonical correlation analysis (CCA) method to investigate the associations between microbiota composition and explanatory variables. Repeated-measures statistical analysis was performed on data for milk microbiome samples at week 1, 4, 8 and 24 of lactation to determine if significant changes occurred over time. To identify the most discriminative taxa that best characterise microbiota composition at week 1, 4, 8 and 24 of lactation, sparse partial least squared-discriminative analysis (sPLS-DA) was conducted using a repeated-measures design on the top 1000 most abundant genera. To determine whether there were bacteria present in the milk microbiota that could discriminate based on mode of delivery, a feature selection statistical analysis LDA effect size (LEfSe), which determines the most likely taxa to explain differences between the groups, was performed at genus level.

### Ethics approval and consent to participate

This study was approved by the Clinical Research Ethics Committee of the Cork Teaching Hospitals, Cork, Ireland (ethical approval reference: ECM (rr) 21/03/17). Participants provided written informed consent, and all relevant guidelines and regulations were followed.

## Results

The aim of this longitudinal study was to investigate how the microbiome evolved in breast milk fed to infants over the first 6 months of life, and to evaluate if these dynamics could be associated with perinatal factors.

### Participants

Breastmilk samples were collected from individuals at 1 week (n = 67), 4 weeks (n = 65), 8 weeks (n = 54) and 24 weeks (n = 42) of lactation. 67% of the participants were studied at three time points and 39% at all four time points. All participants gave birth at full term, and 84% were vaginally delivered and 16% were C-section delivered. Of the infants born, 54% were male and 46% were female. Samples with < 10,000 reads were excluded from the analysis.

### Milk microbiota diversity decreases over lactation

**Figure 1:**
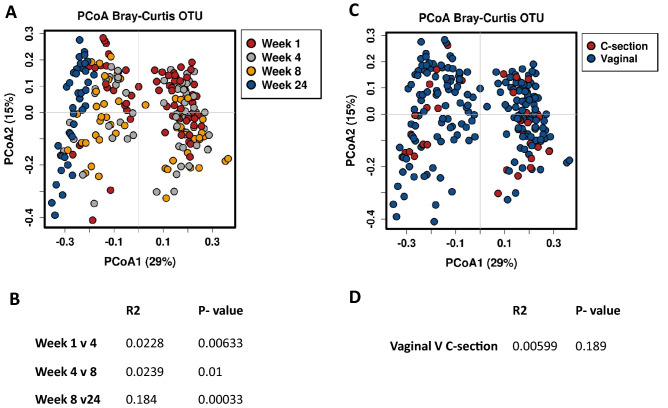
**(A)** PCoA based on Bray–Curtis operational taxonomic unit (OTU) data on effect of lactation stage. (**B)** Adonis variance analysis based on Bray–Curtis distance matrices on lactation stage. **(C)** PCoA based on mode of delivery. **(D)** Adonis variance analysis on mode of delivery.

To determine if any significant differences in beta diversity occurred based on lactation stage, PCoA plots were constructed based on the Bray–Curtis distance matrices at OTU level. Clear separation was observed for week 1 and week 24 samples (Fig. 1A). Adonis variance analysis based on Bray–Curtis distance matrices showed significant difference between all time points (Fig. 1B). No separation was apparent based on birth mode when the PCoA plot was examined, and no significant differences were determined based on Adonis variance analysis (p = 0.189) (Fig. 1C,D). Similarly, analysing samples based on infant sex and maternal BMI showed no clustering or significance (Supplementary Fig. S1).

**Figure 2:**
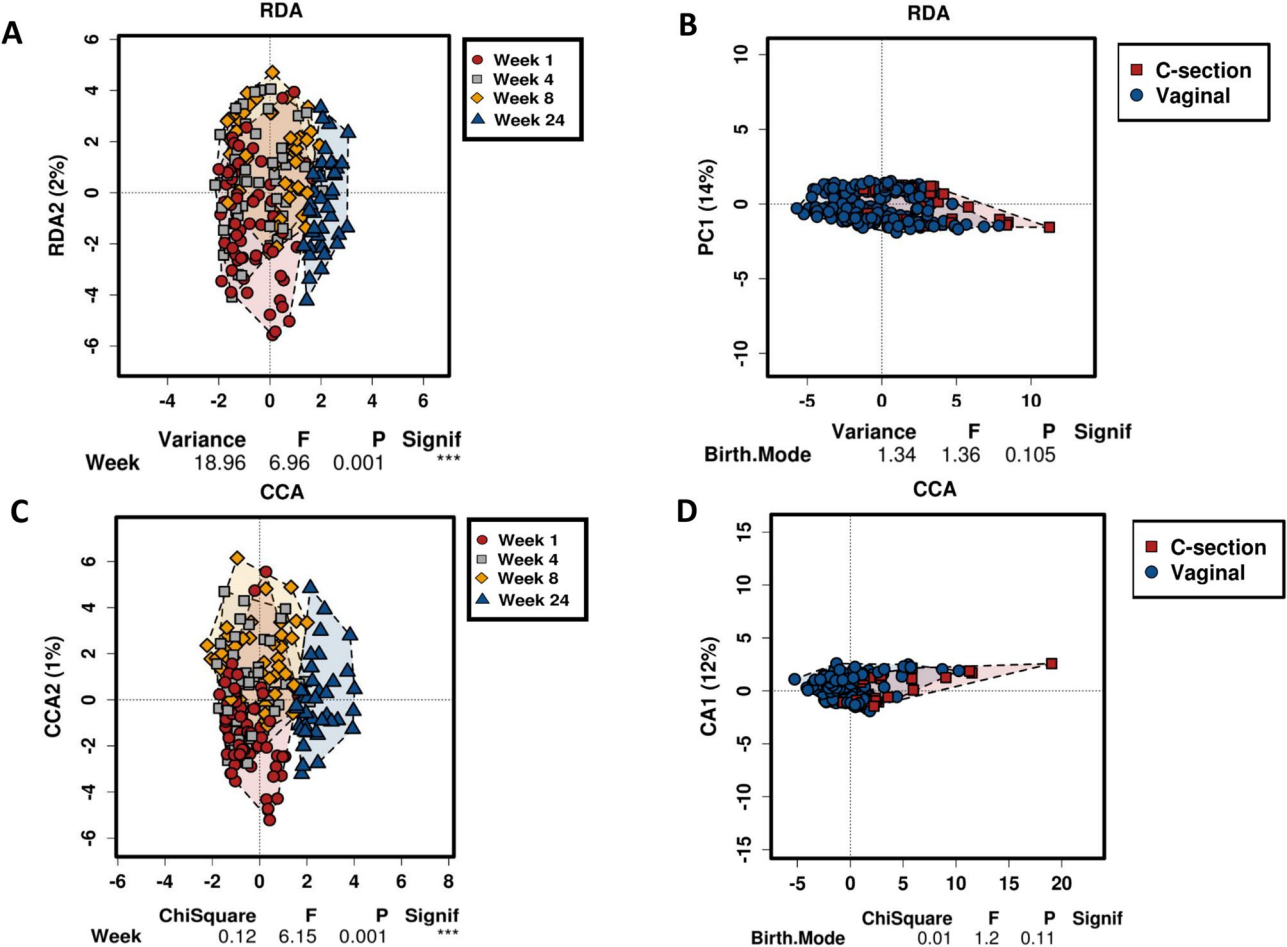
Using a redundancy analysis (RDA) plot and canonical correspondence analysis (CCA) to explore complex associations between community composition and explanatory variables.

Complex associations between community composition and multiple explanatory variables were explored using an RDA plot. We determined that lactation stage had a significant impact on the milk microbiota (p = 0.001) (Fig. 2A), while no significant differences were observed based on birth mode (p = 0.105) (Fig. 2B). Similarly, using CCA, significance was observed based on lactation stage (p = 0.001) (Fig. 2C), while birth mode had no significant impact on the microbiota of milk (p = 0.11) (Fig. 2D) throughout lactation.

**Figure 3:**
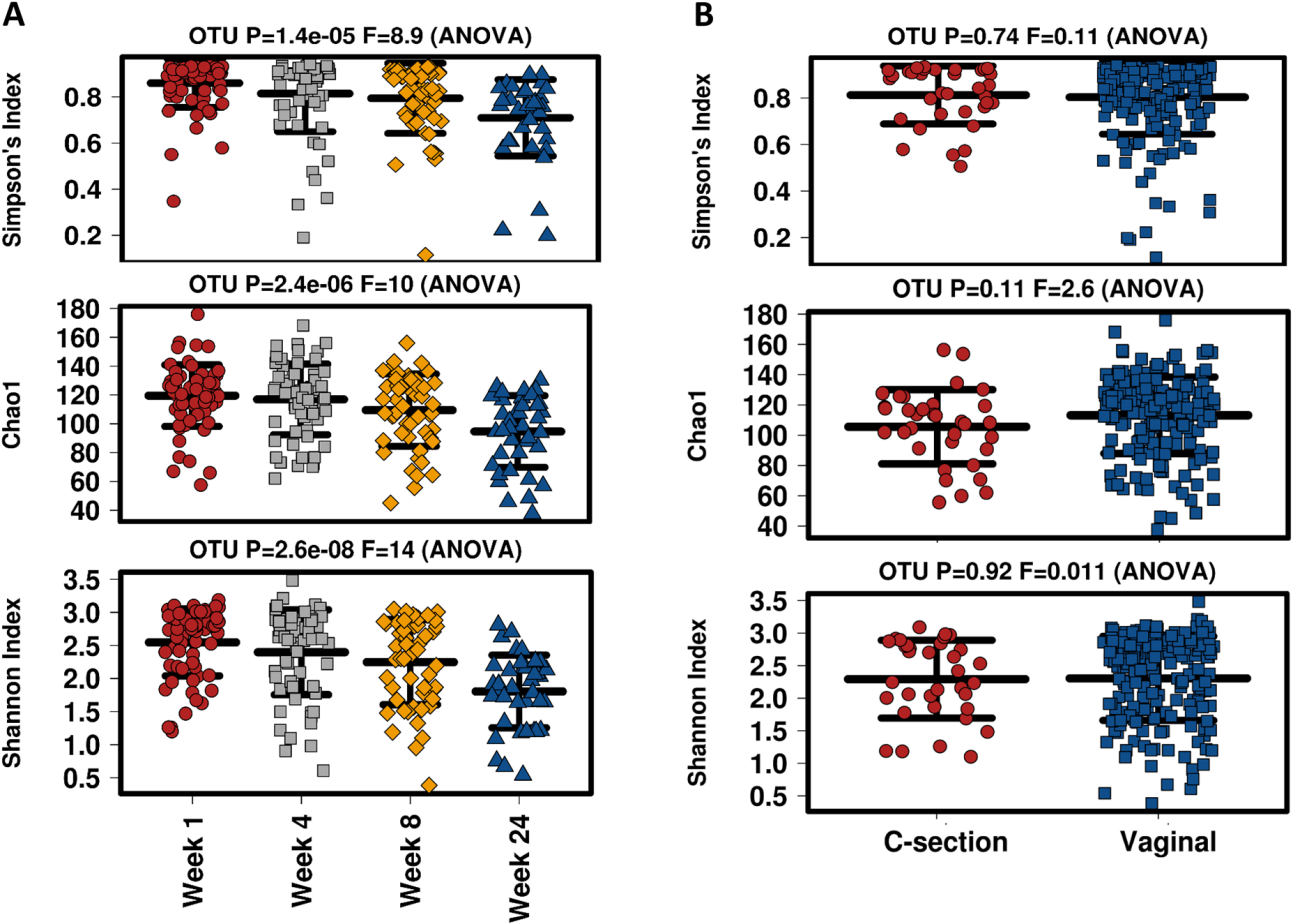
Alpha diversity measures based on lactation stage. Significance observed based on lactation stage across Simpson’s, Chao1 and Shannon indices. No significance in alpha diversity based on mode of delivery.

Alpha diversity significantly decreased over lactation, based on several measures including Shannon Index, Simpson’s Index and Chao1 richness estimator (Fig. 3A). The highest alpha diversity was observed at week 1, with a gradual decrease in diversity over week 4, 8 and 24. The most significant decrease was observed between week 8 and week 24 across all measures. Using the Shannon index, no significant difference was observed between week 1 and 4, and week 4 and 8; however a significant difference was found between week 8 and 24 (p = 0.00062). The Simpson index demonstrated a significant decrease in diversity in milk samples from week 8 to 24 (p = 0.013). Using the Chao1 richness estimator, richness decreased over time, with lowest richness observed at week 24. A significant decrease in richness was detected between week 4 and 8 (p = 0.027) and week 8 and 24 (p = 0.0073) (Table 1). No differences were observed in alpha diversity based on birth mode when all samples (combining vaginal and C-section births) from week 1, 4, 8 and 24 were included (Fig. 3B) (Table 1).

**Table 1:** Alpha diversity measures based on lactation stage and mode of delivery (vaginal VD and caesarean section CS).

	Lactation stage P value				Mode of delivery P value
	Week 1 v 4	Week 4 v 8	Week 8 v 24	Overall	VD v CS
Simpson's index	0.062	0.48	0.013	1.40E−05	0.74
Chao1	0.24	0.027	0.0073	2.40E−06	0.11
Shannon index	0.14	0.2	0.00062	2.60E−08	0.92

**Figure 4:**
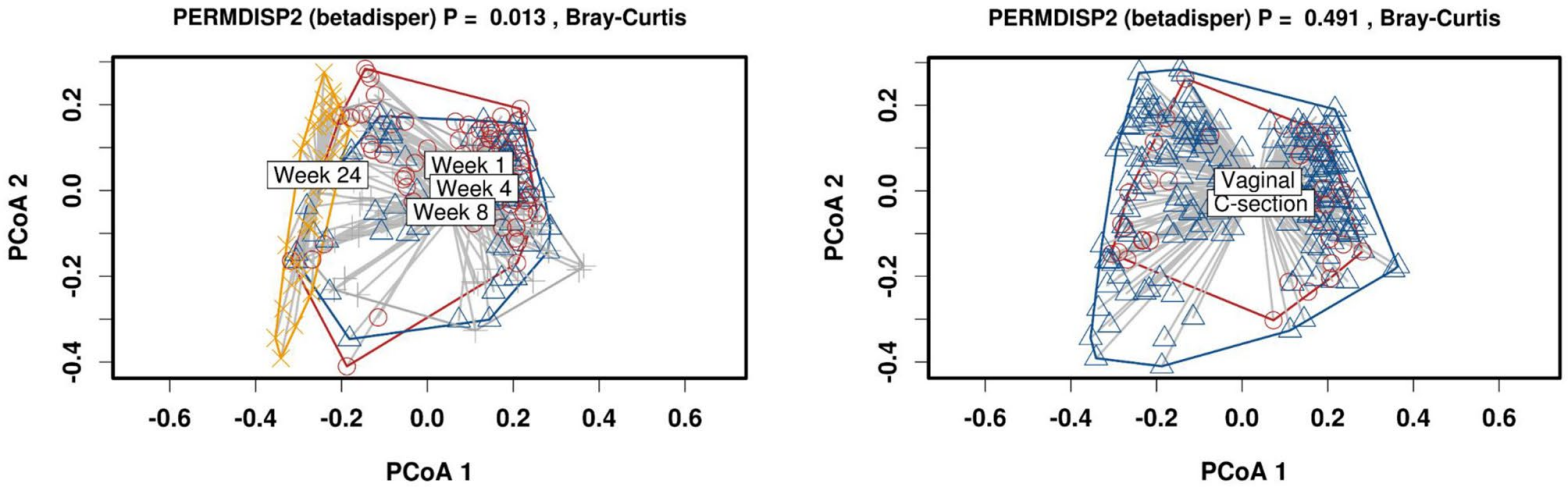
Microbiota composition was significantly different between groups based on lactation stage using PERMDISP2 permutational analysis. P-value is calculated based on distance of each sample to the group centroid in the PCoA and describes significance of the grouping.

To further investigate the factors associated with the clustering of samples based on diversity, principal component analysis (PCA +) was used to map lactation stage and birth mode onto the milk microbiota data. No separation was observed across any time point based on mode of delivery (Supplementary Fig. S2). PERMDISP2 permutational analysis was used to determine whether community composition is significantly different between groups based on the distances of each sample to the group centroid in a principal coordinate analysis. This showed significant separation of samples based on lactation stage (p = 0.013); however, no separation was evident based on birth mode (p = 0.491) (Fig. 4).

**Figure 5:**
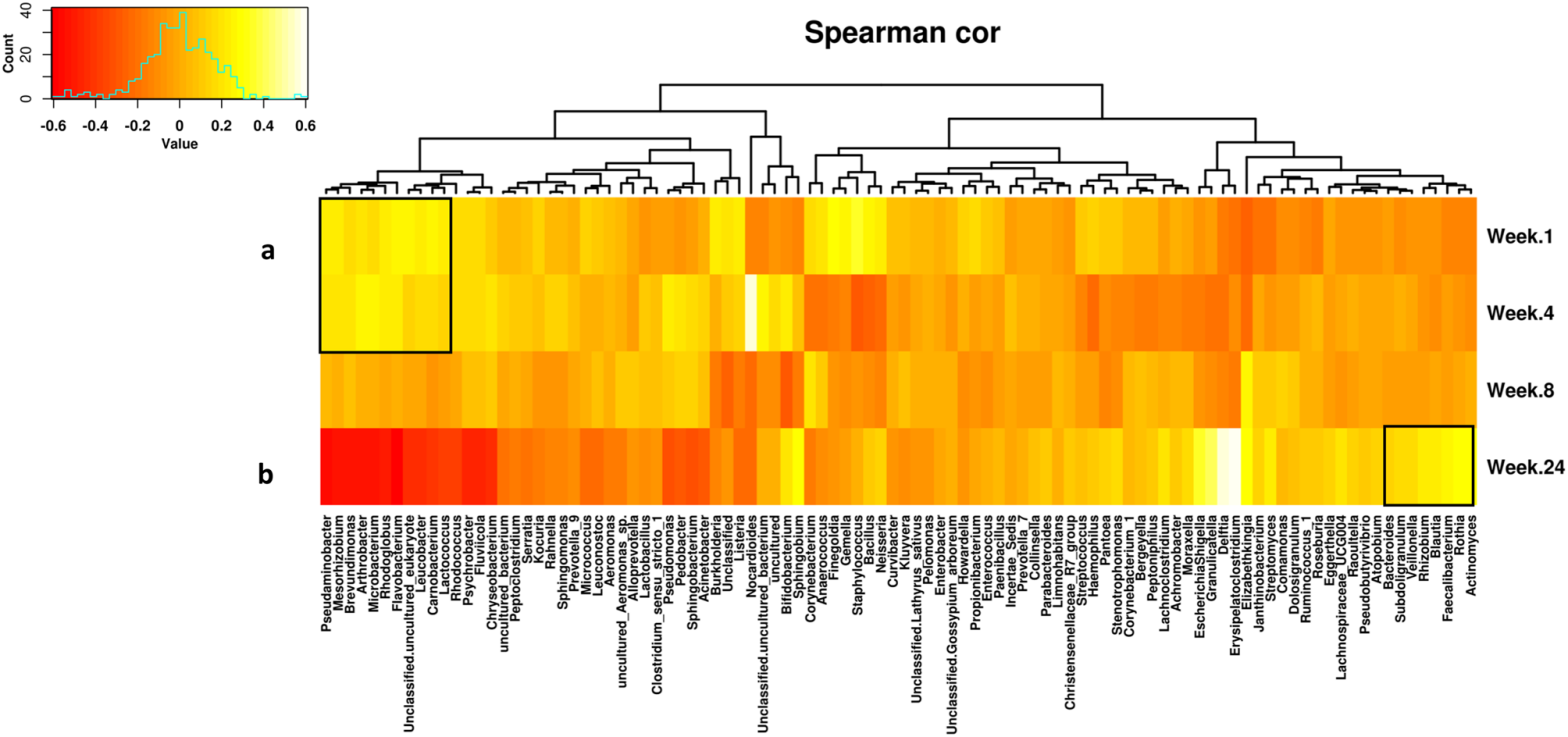
Microbiota associated with lactation stage. Heatmap based on top 100 genera clustered based on lactation time point. Values range from low (red) to high (yellow).

A heatmap was used to determine patterns in the milk microbiota based on lactation stage of the participant. Genera highlighted on the left hand side of the map (A), including *Flavobacterium*, *Brevundimonas* and *Mesorhizobium,* were present in higher abundances at weeks 1 and 4 compared to week 24, whereas genera highlighted on the right of the map (B), including *Actinomyces* and *Veillonella*, were present in higher abundances at week 24 (Fig. 5).

### Milk microbiota composition at week 1, 4, 8 and 24 of lactation

**Figure 6:**
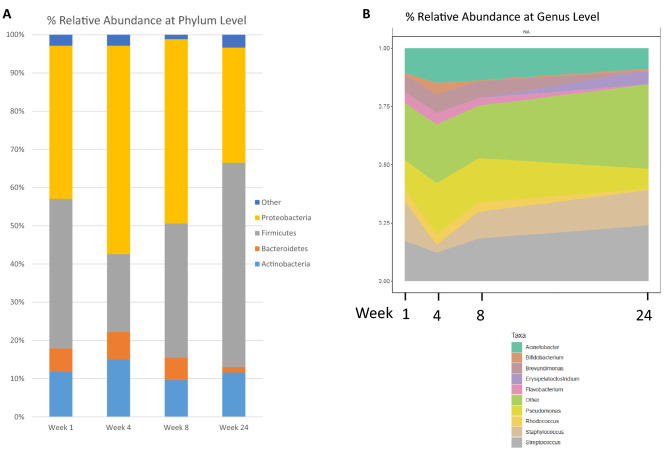
Relative abundances of bacterial **(A)** Phyla and **(B)** Genera over lactation.

There were a number of common phyla shared throughout lactation, with the most prevalent ones found at week 1, 4, 8 and 24 being Proteobacteria, Firmicutes, Bacteroidetes and Actinobacteria. Proteobacteria dominated in early lactation (Week 1–8 40–54%); however, at week 24, Firmicutes dominated at 53% (Fig. 6A).

At genus level, nine genera appeared to predominate throughout the 6 months of lactation. These genera dominated the community, representing 75.5% relative abundance at week 1, 74.8% relative abundance at week 4, 77.5% relative abundance at week 8 and 64% relative abundance at week 24. This core comprised of *Staphylococcus*, *Streptococcus*, *Pseudomonas*, *Acinetobacter, Bifidobacterium, Erysipelatoclostridium, Brevundimonas, Flavobacterium,* and *Rhodococcus* (Fig. 6B). Overall, *Streptococcus* had the highest mean relative abundance at week 1 and 24 (17.3% and 24% respectively), whereas *Pseudomonas* predominated at week 4 (22%) and week 8 (19%). *Bifidobacterium* had the highest mean relative abundance at week 4 of 5% (Fig. 6B). Similarly, *Lactobacillus*, although not identified in the ‘core’ microbiome, had the highest relative abundance at week 4 of 1.4%. The relative abundance of these genera was individual specific and subject to intra-individual variations over time (Supplementary Fig. S3).

To explore the changes and evolution of the milk microbiota of women between 1, 4, 8 and 24 weeks of lactation, repeated-measures statistical analysis was performed on the data. Individuals who provided milk samples at more than one time point were included in the analysis. At phylum level, Proteobacteria (p < 0.01), Firmicutes (p < 0.001), Bacteroidetes (p < 0.001), Actinobacteria (p < 0.05) were all significantly different in abundance between time points (Supplementary Table S2). At the genus level, 49 significant differences were observed between week 1 and week 24, including a significant increase in *Bifidobacterium* (p < 0.001), *Veillonella* (p < 0.01), *Blautia* (p < 0.001) and *Faecalibacterium* (p < 0.001), and a significant decrease in *Pseudomonas* (p < 0.01), *Acinetobacter* (p < 0.001), *Pseudaminobacter* (p < 0.001), and *Flavobacterium* (p < 0.001) (Table 2). Although most significant changes were observed between week 1 and week 24, significant fluctuations in genera were observed between all time points. *Bifidobacterium* significantly increased (p < 0.01) and *Staphylococcus* significantly decreased (p < 0.01) from weeks 1 to 4. *Bifidobacterium* significantly decreased from weeks 4 to 8, and weeks 8 to 24 (p < 0.001) (Table 3).

**Table 2:** Repeated measures statistical analysis between Week 1 and Week 24 milk samples at genus level.

Taxa	Week P. bonferroni	Week FDR p-value	Week coefficient
*Flavobacterium*	0	< 0.001	−11
*Pseudaminobacter*	3.5E−20	< 0.001	−9.4
*Brevundimonas*	8.9E−20	< 0.001	−9.3
*Mesorhizobium*	6.9E−18	< 0.001	−9.1
*Arthrobacter*	1.5E−17	< 0.001	−8
*Microbacterium*	9.6E−17	< 0.001	−8.2
*Rhodoglobus*	2.4E−16	< 0.001	−7.7
*Psychrobacter*	2.4E−13	< 0.001	−7
*Fluviicola*	2.9E−12	< 0.001	−5.8
*Leucobacter*	4.7E−12	< 0.001	−5.4
*Carnobacterium*	2.4E−10	< 0.001	−4.2
*Chryseobacterium*	1.4E−09	< 0.001	−5.1
*Delftia*	2.8E−08	< 0.001	4
*Rhodococcus*	5.7E−08	< 0.001	−5.1
*Erysipelatoclostridium*	2.6E−07	< 0.001	4.3
*Lactococcus*	3.3E−07	< 0.001	−3.3
*Elizabethkingia*	0.0000014	< 0.001	3.5
*Granulicatella*	0.0000041	< 0.001	4.8
*EscherichiaShigella*	0.000047	< 0.001	3.7
*Actinomyces*	0.000063	< 0.001	4
*Faecalibacterium*	0.000078	< 0.001	2.6
*Rothia*	0.00077	< 0.001	4
*Sphingobium*	0.0014	< 0.001	2.8
*Listeria*	0.0015	< 0.001	−2.6
*Blautia*	0.0074	< 0.001	2
*Acinetobacter*	0.0086	< 0.001	−3.2
*Bifidobacterium*	0.012	< 0.001	2
*Pseudomonas*	0.029	< 0.01	−2.7
*Subdoligranulum*	0.03	< 0.01	1.9
*Sphingobacterium*	0.041	< 0.01	−2.5
*Serratia*	0.042	< 0.01	−2.3
*Rhizobium*	0.045	< 0.01	2.5
*Pedobacter*	0.055	< 0.01	−1.7
*Leptotrichia*	0.064	< 0.01	1.9
*Atopobium*	0.068	< 0.01	1.7
*Corynebacterium*	0.1	< 0.01	−1.8
*Ruminococcus_1*	0.11	< 0.01	1.6
*Veillonella*	0.14	< 0.01	2.4
*Gemella*	0.14	< 0.01	−2.1
*Lachnoclostridium*	0.19	< 0.01	1.5
*Bacteroides*	0.23	< 0.05	1.6
*Kocuria*	0.28	< 0.05	−1.5
*Lachnospiraceae_UCG004*	0.33	< 0.05	1.5
*Anaerococcus*	0.37	< 0.05	−1.6
*Staphylococcus*	0.44	< 0.05	−1.6
*Moraxella*	0.51	< 0.05	1.7
*Pseudobutyrivibrio*	0.58	< 0.05	1.2
*Finegoldia*	0.64	< 0.05	−1.6
*Stenotrophomonas*	0.92	< 0.05	1.7

**Table 3:** Repeated measures statistical analysis over time at genus level.

Taxa	Week P. bonferroni	Week FDR p-value	Week coefficient
**Week 1 and Week 4**			
*Staphylococcus*	0.0000012	< 0.001	−2.9
*Bacillus*	0.0018	< 0.001	−2.2
*Gemella*	0.0038	< 0.001	−2.2
*Anaerococcus*	0.031	< 0.01	−1.6
*Bifidobacterium*	0.033	< 0.01	1.9
*Haemophilus*	0.038	< 0.01	−2.3
*Lactobacillus*	0.1	< 0.05	1.6
*Sphingobacterium*	0.36	< 0.05	1.4
**Week 4 and Week 8**			
*Blastocatella*	1.9E−12	< 0.001	−3.6
*Nocardioides*	2E−10	< 0.001	−3.3
*Elizabethkingia*	0.0000066	< 0.001	2.5
*Bifidobacterium*	0.00068	< 0.001	−3.1
*Listeria*	0.014	< 0.01	−1.9
*Sphingomonas*	0.11	< 0.05	−1.6
**Week 8 and Week 24**			
*Flavobacterium*	2.2E−16	< 0.001	9.3
*Pseudaminobacter*	7.8E−13	< 0.001	7.8
*Mesorhizobium*	3.3E−11	< 0.001	7.6
*Arthrobacter*	1E−10	< 0.001	6.6
*Brevundimonas*	1.4E−10	< 0.001	7.7
*Microbacterium*	1.6E−09	< 0.001	6.9
*Rhodoglobus*	8.4E−09	< 0.001	6
*Psychrobacter*	6.4E−08	< 0.001	6.1
*Granulicatella*	0.0000014	< 0.001	−5.2
*Bifidobacterium*	0.0000061	< 0.001	−3.1
*Chryseobacterium*	0.000015	< 0.001	4.8
*Sphingobacterium*	0.000055	< 0.001	4.2
*EscherichiaShigella*	0.00022	< 0.001	−3.8
*Pseudomonas*	0.0011	< 0.001	3.6
*Acinetobacter*	0.0015	< 0.001	3.7
*Faecalibacterium*	0.0063	< 0.001	−2.1
*Stenotrophomonas*	0.0071	< 0.001	−3.1
*Actinomyces*	0.0082	< 0.001	−3
*Rhodococcus*	0.0096	< 0.001	3.4
*Rothia*	0.031	< 0.01	−2.9
*Blautia*	0.048	< 0.01	−1.6
*Bacteroides*	0.26	< 0.05	−1.4
*Veillonella*	0.28	< 0.05	−2.2
*Subdoligranulum*	0.32	< 0.05	−1.3

### Identifying discriminative milk taxa at 1, 4, 8, and 24 weeks of lactation

**Figure 7:**
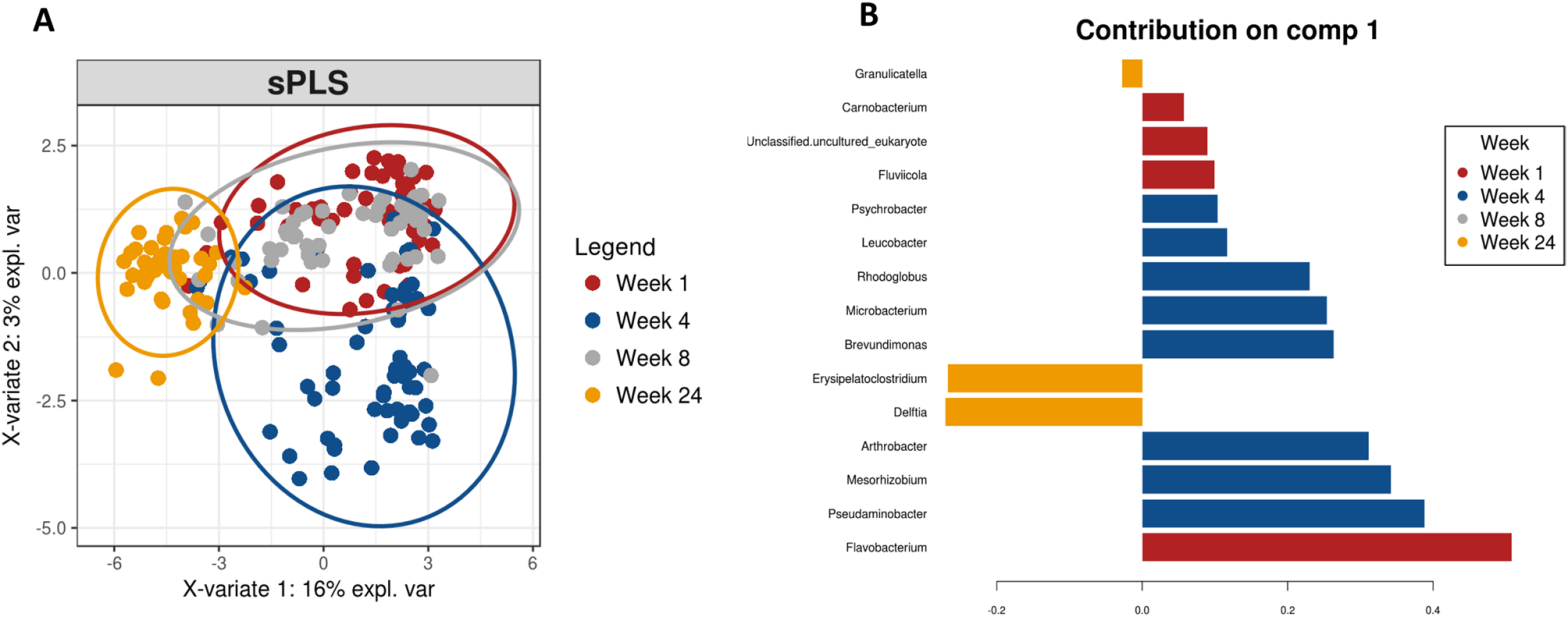
**(A)** Sparse Partial Least Squared–Discriminative Analysis plot demonstrating a clear separation on repeated measures over lactation between week one, week four, week eight and week 24 using the top 1000 most abundant genera. (**B)** Contribution chart highlighting taxa associated with each time point.

To identify the most discriminative taxa that best characterise the milk microbiota composition at 1, 4, 8 and 24 weeks of lactation, sPLS-DA was performed using a repeated-measures design on the top 1000 most abundant genera present at ≥ 2 time points. *Flavobacterium* was best found to characterise the microbiota profile at week 1, *Arthrobacter, Mesorhizobium* and *Pseudaminobacter* at week 4 and *Erysipelatoclostrium* and *Delfia* at week 24 (Fig. 7A,B).

### Identifying discriminative taxa based on mode of delivery

In order to determine whether birth mode exerted measurable effects on the milk microbiota at weeks 1, 4, 8 and 24 of lactation, ANalysis of COmposition of Microbiomes (ANCOM) was performed on the top 1000 most abundant taxa at the phylum, family and genus levels. No significant differences were observed across lactation using ANCOM between those who gave birth vaginally or by C-section. Similarly, the Mann–Whitney test resulted in no significant differences between the two groups.

**Figure 8:**
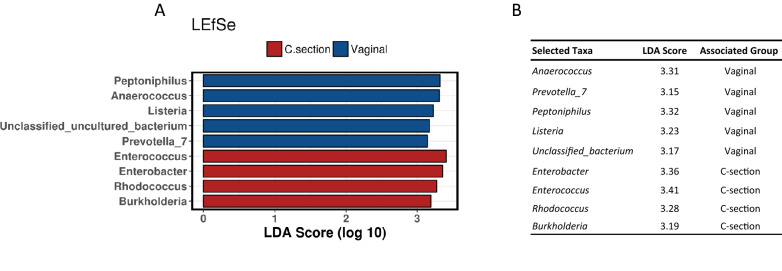
LEfSe analysis determining discriminative taxa based on mode of delivery at genus level **(A)** and corresponding LDA (discriminatory power) values **(B)**.

To determine whether there were bacteria present in the milk microbiota that could discriminate based on mode of delivery at birth, we used LEfSe which determines the features (in this study, genera) most likely to explain differences between groups. Overall, nine genera discriminated the milk samples from women who gave birth vaginally and via C-section. Vaginal deliveries were characterised by *Peptoniphilis, Anaerococcus*, *Listeria* and an unclassified bacterium, whereas C-section deliveries were associated with *Enterococcus, Enterobacter, Rhodoccus* and *Burkholderia* (Fig. 8A). *Peptoniphilis* and *Anaerococcus* had the highest power for indication of vaginal deliveries (LDA 3.32, 3.31 respectively) while *Enterococcus* and *Enterobacter* had the highest discriminatory power for C-section birth mode (LDA 3.41, 3.36 respectively) (Fig. 8B).

To determine if the microbiota present could discriminate mode of delivery at each stage of lactation, LEfSe was performed between vaginal and C-section births at week 1, 4, 8 and 24. At week 1 of lactation, two genera, *Peptoniphilus* and *Abiotrophia*, discriminated those who gave birth vaginally from those that had C-section. No genera were observed to discriminate for C-section births at week 1 of lactation. At week 4 of lactation, six genera discriminated those who gave birth vaginally or by C-section. Vaginal birth samples were discriminated by *Moraxella, Propionibacterium* and *Listeria*, while C-section birth samples were associated with *Sphingobacterium, Enterobacter* and *Rhodococcus*, with *Sphingobacterium* and *Enterobacter* having the greatest discriminatory power for C-section birth mode. At 8 weeks of lactation, several discriminative bacteria were identified between women based on birth mode, with *Pedobacter* having the highest discriminatory power for vaginal births and *Bifidobacterium* having the highest discriminatory power for C-section. At 24 weeks of lactation, five discriminative bacteria were found at genus level, with *Stenotrophomonas* and *Bacillus* identified in milk of women who gave birth by vaginal delivery, while *Burkholderia* and *Brevundimonas* were associated with C-section deliveries (Supplementary Fig. S4).

## Discussion

Much research now exists concerning the bacterial communities present in human milk and the various factors that affect microbial composition in early lactation^10–12,15,19,20,32,33^. However, data on what factors, if any, continue to exert measurable effects on human milk microbiota throughout lactation are limited. Thus, this large-scale longitudinal study investigated the evolution of the milk microbiome throughout lactation over 6 months, while also examining whether factors such as mode of delivery, maternal BMI and infant gender would be reflective in the milk microbiota up to 6 months postpartum.

In line with existing data, our study confirms the presence of a milk microbiome which is highly individual specific and subject to intra-individual variations over time^19,33^. This longitudinal study allowed for the analysis of the evolution of the breast milk microbiota over lactation taking mode of delivery into account. Previous studies have reported that the microbial composition of breast milk changes over time from colostrum to transitional to mature milk^14^, and mode of delivery has also been determined to be associated with changes to milk bacterial communities^32^. We identified a significant decrease in milk microbiota diversity throughout lactation from birth to 6 months, with the greatest significant difference observed between week 8 and week 24 of lactation. A decrease in diversity and richness over time has been observed and reported previously^19,20,34^, and may be linked to changing nutritional composition of the milk over lactation. As reported previously, milk is a dynamic living tissue and changes in terms of nutritional composition and bioactive compounds throughout lactation to meet the needs of the developing infant^35^. The higher microbial diversity detected in early lactation could be due to the higher presence of nutrients such as proteins, and bioactives such as immunoglobulins, cytokines and HMOs in colostrum and early milk^36,37^. Investigations have revealed some correlations between milk microbiota profiles and HMOs^38,39^. This change in microbiota composition and diversity may also be attributed to the developing infant and maturing infant microbiota. However, it is worth noting that although a decrease in milk microbiota diversity was observed in this study, the infant gut microbiome typically increases in diversity during the same time interval, suggesting that the increase in microbiota diversity in the infant gut is not coming directly from the bacteria present in milk.

Interestingly, no difference in diversity was observed between milk samples from women who gave birth vaginally compared to milk samples from women who gave birth via C-section. This finding has also been reported in other studies^40–42^.

The abundance of several phyla differed significantly with stage of lactation. Firmicutes significantly increased over time while Proteobacteria significantly decreased from week 4 to 24 lactation. This is in line with other studies that have reported that Firmicutes and Proteobacteria dominate in milk samples, but their proportions change considerably depending on sampling week^43^.

At genus level, a core of nine genera were identified in our study, with *Staphylococcus, Streptococcus*, *Pseudomonas* and *Acinetobacter* being present in the highest mean relative abundances at all time points. Previous studies have documented different core bacterial profiles; however, *Staphylococcus, Streptococcus* and *Pseudomonas* have been consistently reported as key genera in the milk microbiota^19,41–44^. While no significant changes occurred in *Streptococcus* over time, *Staphylococcus* significantly decreased from week 1 to week 4 while the opposite was observed for *Pseudomonas* which significantly increased. Furthermore, it has been reported that the gut of new-born infants is colonised by high abundances of *Staphylococcus*, followed by a sharp decrease after the first week of life^19,45^. This is consistent with reports that vertical transmission occurs from mother to infant, with breast milk seeding the infant gut with microbial populations. While a similar finding was observed by Murphy et al. who reported a decrease in *Staphylococcus* up to 12 weeks^19^ and Wan et al. who documented a decrease up to 6 weeks^34^, our longitudinal study revealed a gradual increase from week 4 to 6 months postpartum. It is also important to mention that mastitis, a bacterial infection resulting in inflammation of the breast tissue is a leading cause of breastfeeding cessation and premature weaning. Mastitis can be classified into clinical or subclinical, with *Staphylococcus aureus* identified as the main causative agent of clinical mastitis, and *Staphylococcus epidermidis* the causative pathogen of subclinical mastitis. Interestingly, a recent study by our group (data not published) examined milk from healthy lactating women and milk from women with mastitis and found that over one third of the milk samples from healthy women had a high somatic cell count and IL-8 levels, indicating potential subclinical/asymptomatic mastitis. This suggests that there may be a high prevalence of undetected subclinical mastitis across the lactating population due to the possible asymptomatic nature of the infection, which may have implications for both maternal and infant health. Although the milk in this study was analysed from healthy women, it is possible that a proportion of the participants may have had asymptomatic, subclinical mastitis and this in turn may account for the increase in *Staphylococcus* observed.

It is also worth noting that *Acinetobacter* was prevalent across all time points in our study, and this genus has been commonly detected in many milk microbiome studies^15,46,47^. However, in our study, we observed that, as the relative abundance of *Staphylococcus* increased, that of *Acinetobacter* decreased and vice versa. A similar finding was reported by Li et al. who reported that *Staphylococcus* had a negative correlation with *Acinetobacter*^15^. Additionally, Patel et al. investigated the milk from healthy controls and participants with subacute and acute mastitis and noted that *Acinetobacter* was consistently depleted in those with subacute and acute mastitis, while *Staphylococcus* was enriched^46^.

Although not identified in the core milk microbiome in this study, the genera *Actinomyces, Veillonella, Rothia* and *Prevotella* were present in higher relative abundances (> 1%) at week 24 lactation. These genera, including *Streptococcus*, are associated with the infant oral microbiome^48–50^, and this supports the rationale that the infant oral cavity is a source of bacteria for breast milk, thus supporting the hypothesis of retrograde backflow during infant suckling, resulting in the backflow of saliva from the infants oral cavity into the mammary gland^35^. Moreover, it has been reported that the milk microbiome of women following preterm births contained lower levels of *Streptococcus* compared to full term births. This is reportedly due to very low birth weight infants receiving milk via feeding tubes and not via the breast^51^. Other studies have reported an increase in *Streptococcus* following latching of the infant to mothers breast^52^.

Potential probiotic species belonging to the genera *Bifidobacterium* and *Lactobacillus* are the focus of many milk culturing and isolation studies due to their health promoting benefits and potential in the infant nutrition market. While some investigations have identified these genera as part of the core milk microbiome^19,42,53^, *Bifidobacterium* and *Lactobacillus* were present in low abundances in our study, both having the highest mean relative abundance at week 4, but present at ≤ 1% at all other time points. Previous studies have also noted low abundances of *Bifidobacterium* and *Lactobacillus* in milk^40^. *Bifidobacterium* and *Lactobacillus* have been reported as part of the infant gut core microbiota, with *Bifidobacterium* in particular being one of the most prevalent microbes present in the new-born infant gut^19,54–56^.

Gut-associated microbes such *as Bifidobacterium, Erysipelatoclostridium*, and *Enterobacter* were detected in the milk in this study, albeit at low levels, with *Erysipelatoclostridium* and *Enterobacter* having the highest relative abundance at 6 months of lactation. The presence of anaerobic gut-associated microbes in milk further supports evidence of an entero-mammary pathway which allows for the transfer of bacteria from maternal gut to the mammary gland. A recent study by Kordy et al. identified a distinct *Bifidobacterium breve* strain in the mother’s rectum, breast milk, and subsequent infant gut. The results of this study are consistent with the presence of an entero-mammary pathway, a retrograde mechanism of milk inoculation, which allows for the transfer of bacteria such as *Bifidobacterium breve* from maternal gut up to the mammary gland by an immune cell-mediated bacterial translocation^57^.

Although no significant differences were observed for diversity and taxa in the milk microbiome based on mode of delivery, some discriminate taxa were identified between women who gave birth vaginally and via C-section. The milk of women who gave birth via C-section had more *Enterobacter* and *Enterococcus* than of those who delivered vaginally (although differences were not significant) and it has been reported that the gut microbiota of infants following C-section deliveries is colonised by higher levels of *Enterococcus* and *Enterobacter,* further supporting a potential correlation between milk and gut microbiomes^58,59^. As C-section deliveries are increasing globally, it is imperative that we understand the true impact of these perinatal factors on the milk microbiome and subsequently the infant gut^60^.

While mode of delivery exerted no impact on milk microbiome composition in our study, and several other studies^40–42,61^, it has been reported that milk samples from women with non-elective C-sections were more similar to those from women with vaginal deliveries than milk samples from women with elective C-sections, suggesting that physiological stress during delivery may play a role in the transfer of bacteria to breast milk^20^. Many non-elective C-sections deliveries are also preceded by failed invasive instrumental delivery attempts (forceps or vacuum) which might be also contributory. It is widely accepted that mode of delivery impacts the infant gut microbiota; however, due to conflicting reports, more detailed investigations involving larger cohorts are needed to determine the true impact, if any, of mode of delivery on the milk microbiome.

Conflicting reports on the impact of maternal BMI and weight gain on milk microbiota composition have been reported across the literature. Our study revealed no impact of maternal BMI on milk microbiome diversity. This finding was also reported across several other studies^16,33,62^. However, Cabrera-Rubio et al. reported that maternal BMI and weight gain during pregnancy had an impact on bacterial composition and diversity in breast milk, with high BMI associated with a decrease in diversity. These differences across studies may be attributed to a number of factors such as inter-individual variation, geographical location and diet culture, differing scientific approaches and the small sample size (n = 18)^20^. In conclusion, the results of this study indicate that stage of lactation is the primary driving factor influencing the human milk microbiome, while mode of delivery does not impact changes in the composition of the microbiome that occur in milk throughout human lactation up to 6 months. While there have been previous analyses of the milk microbiota composition over lactation, showing shifts in microbial composition due to factors such as gestational age, antibiotic usage, maternal health and diet, infant sex and geographical location^18,33,41,63^, the present study further expands on these findings, documenting the evolution of the milk microbiome up to 6 months of lactation while also taking mode of delivery into account.

## Supplementary Information

Supplementary Information.

## Data Availability

Data available upon request.
