# Supplementary material for: The human milk microbiome aligns with lactation stage and not birth mode
Source: Sci Rep. 2022 Apr 4;12:5598. doi: 10.1038/s41598-022-09009-y (PMC8979980; doi:10.1038/s41598-022-09009-y)
Supplement: Supplementary file 1 — Supplementary Information. [file 41598_2022_9009_MOESM1_ESM.docx]

**Supplementary Information**

The human milk microbiome aligns with lactation stage and not birth mode

**B**

**A**


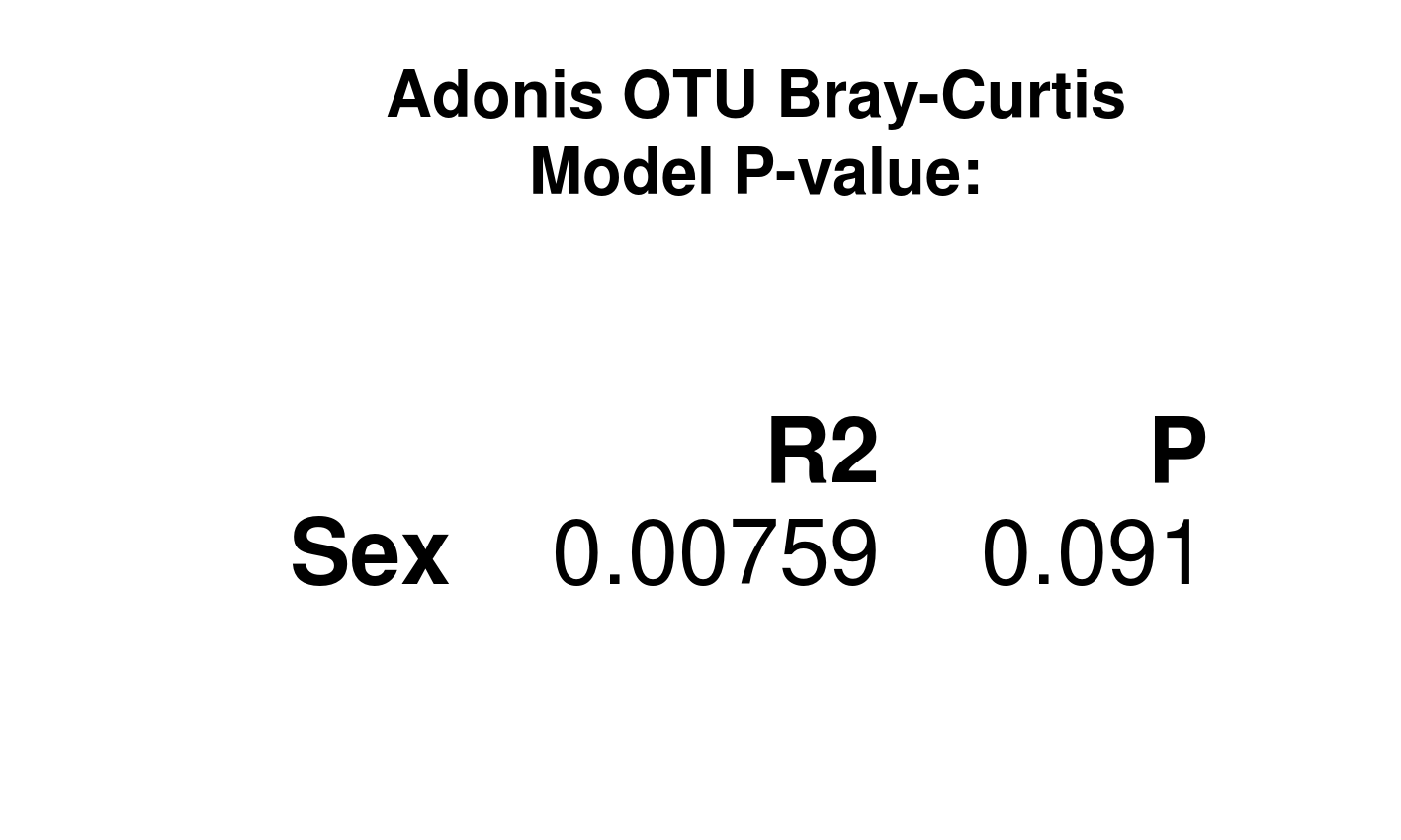

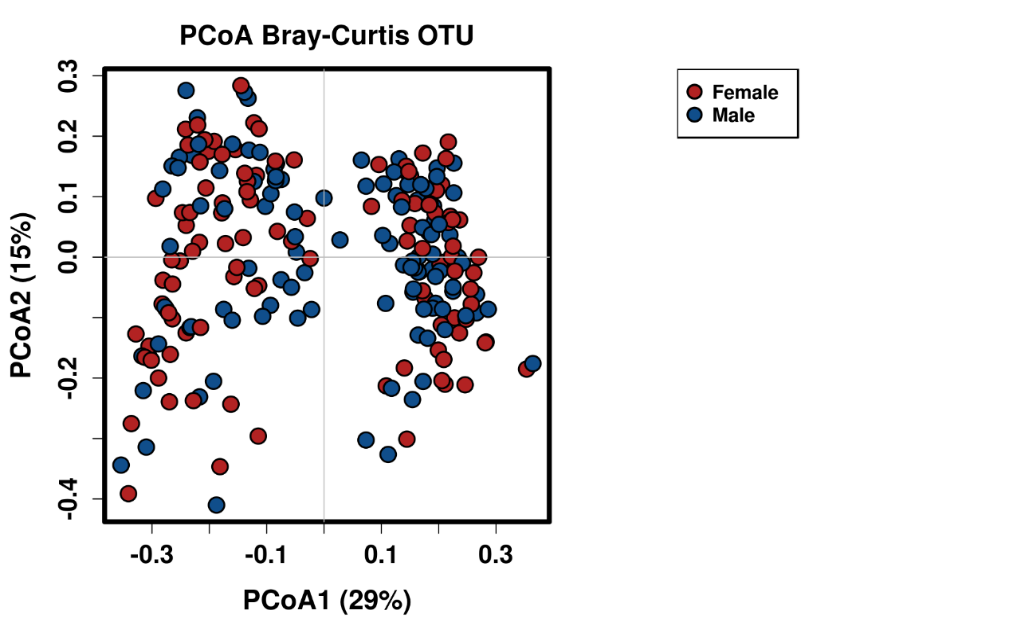

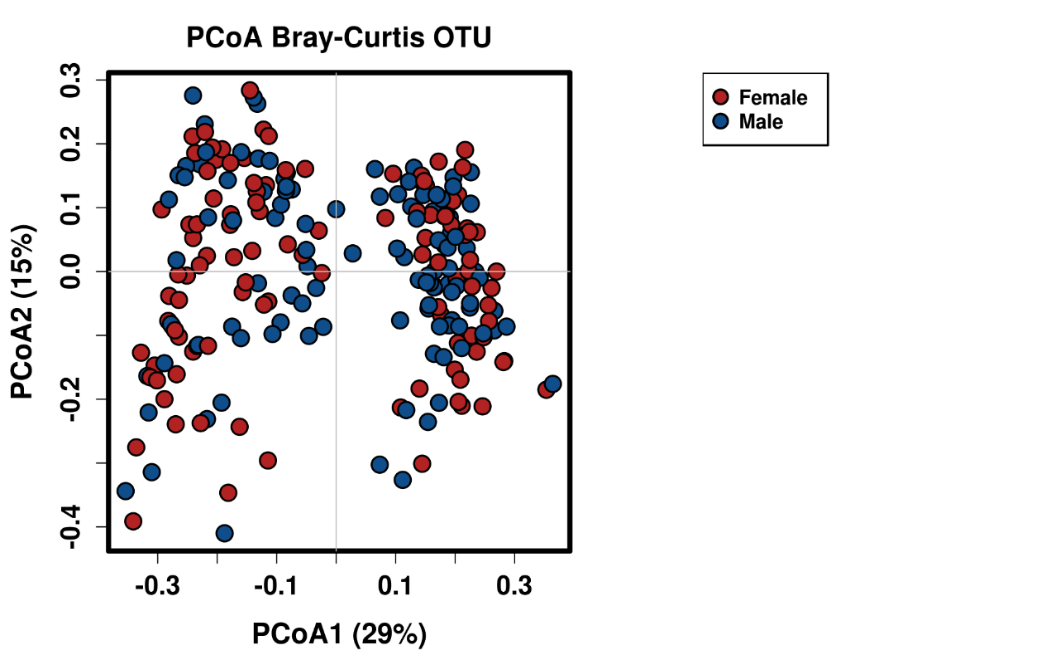


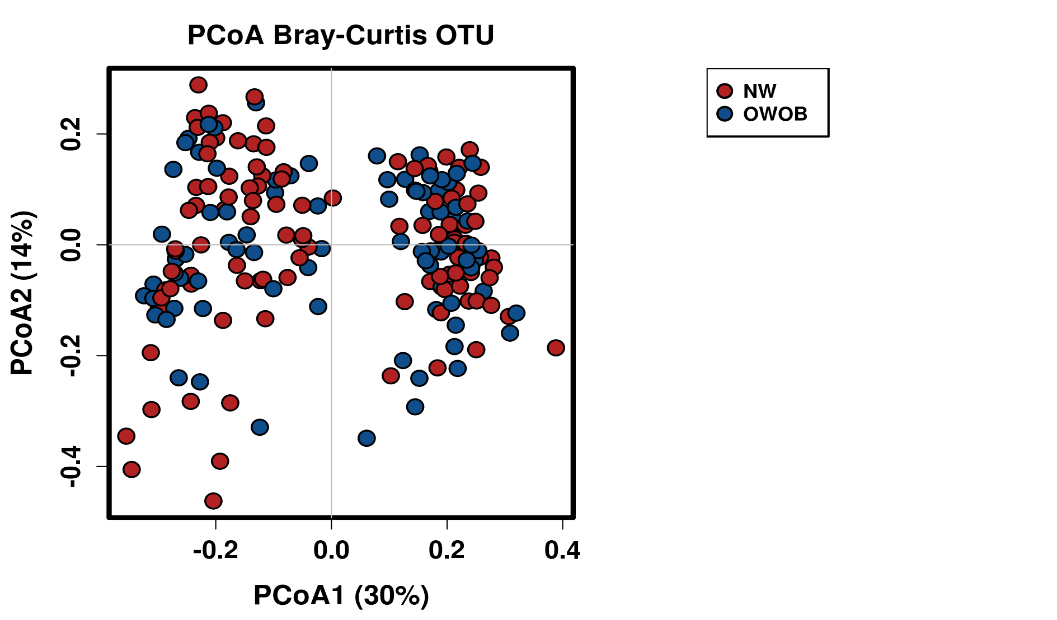

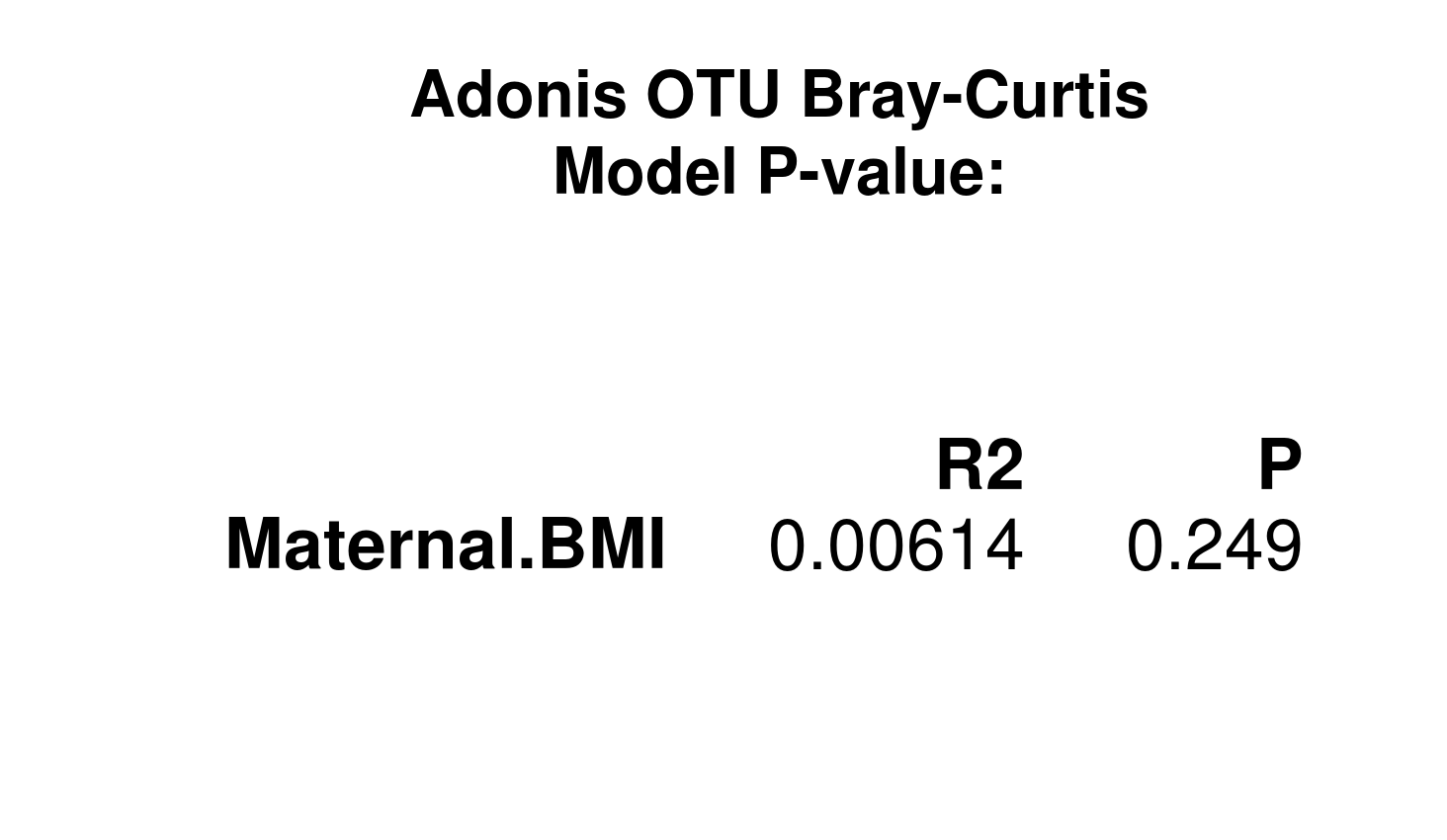

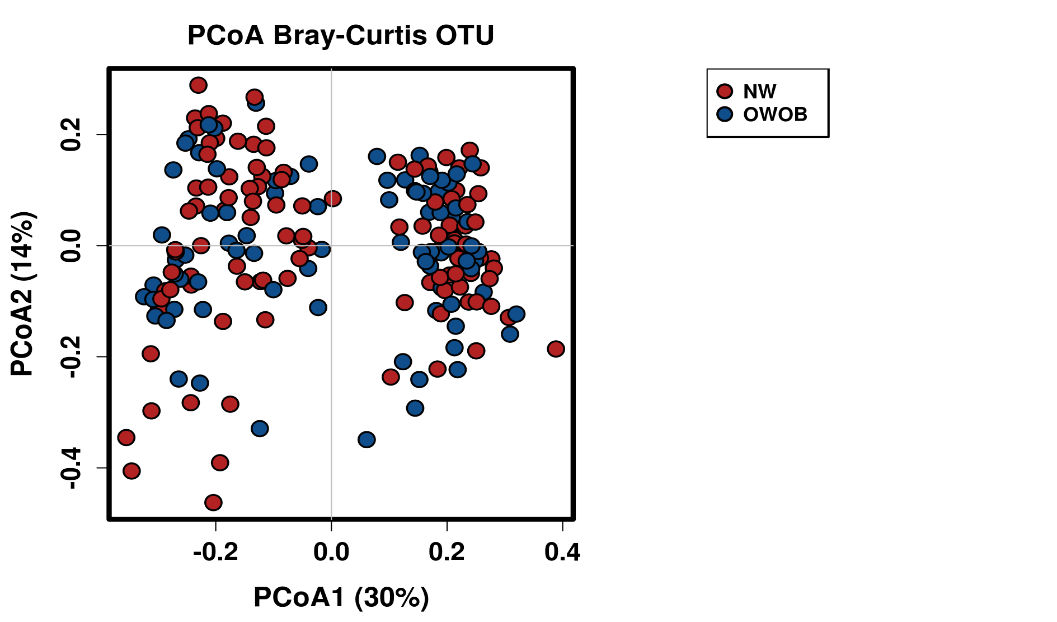


**Supplementary Figure S1.** PCoA analysis using Bray-Curtis distance matrices based on; **A** Infant Sex and **B** Maternal BMI (NW (normal weight) = BMI <25, OW/OB (overweight/obese) = BMI >25). Adonis variance analysis showed no significance based on infant sex or maternal BMI.

**B**

**A**


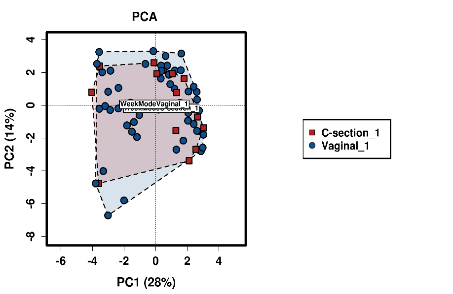

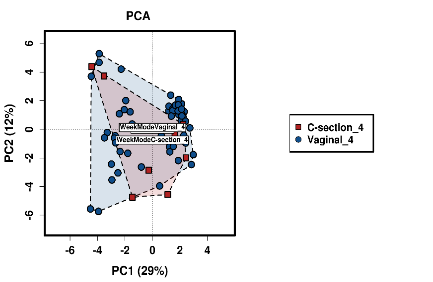

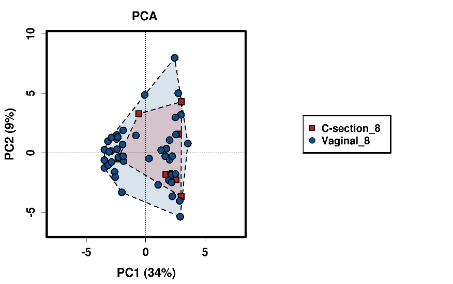

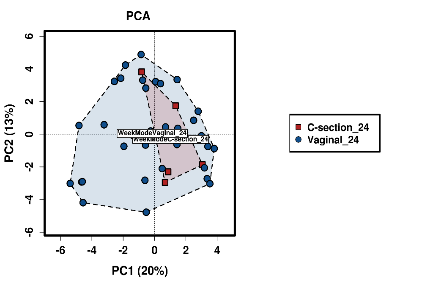


**Supplementary Figure S2**: Principal component analysis (PCA+) mapping lactation stage and birth mode onto milk microbiota data. No separation occurred between vaginally or C-section across the different time points. **A** vaginal and c-section at week 1; **B** vaginal and c-section at week 4; **C** vaginal and c-section at week 8; **D** vaginal and c-section at week 24

**D**

**C**


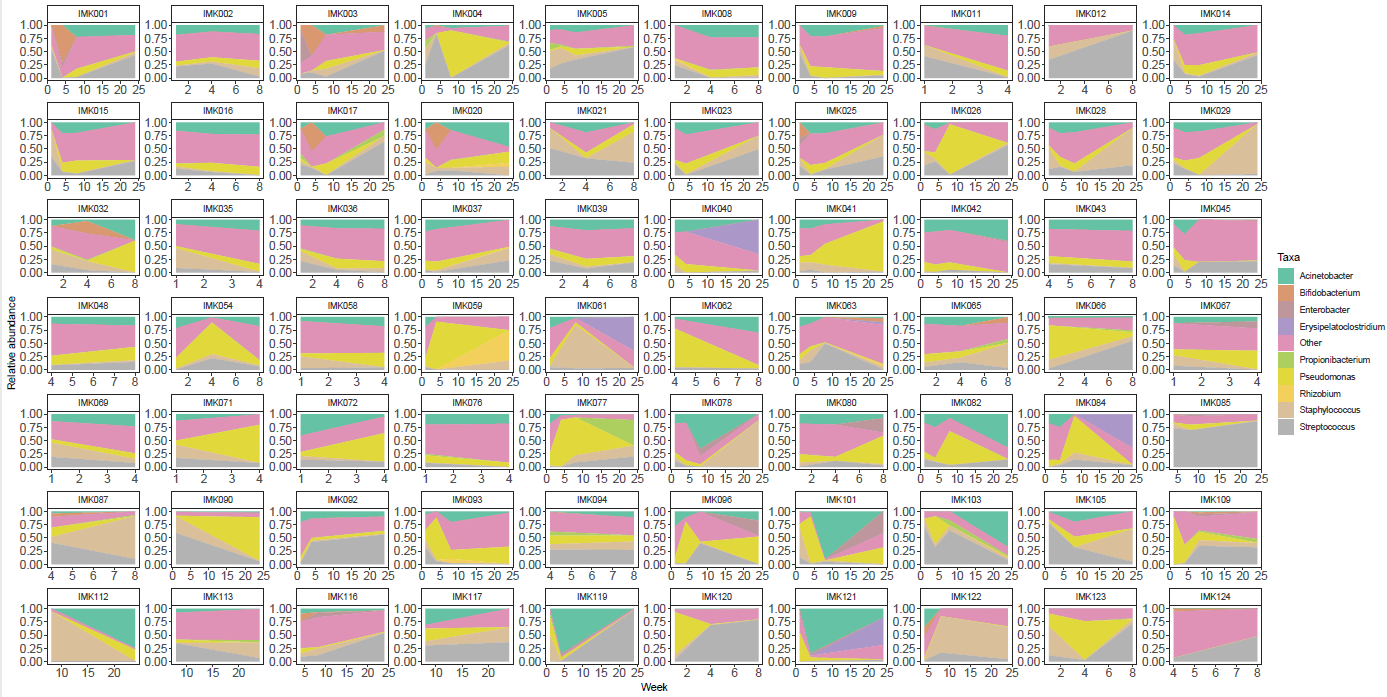


**Supplementary Figure S3**. Sankey plot of relative abundance at genus level. The relative abundances of these genera were individual specific and subject to intra-individual variations over time.


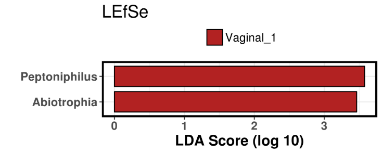

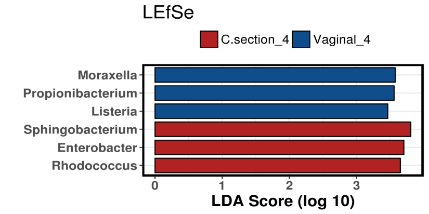

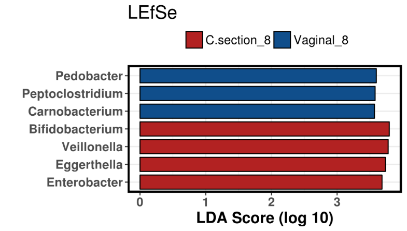

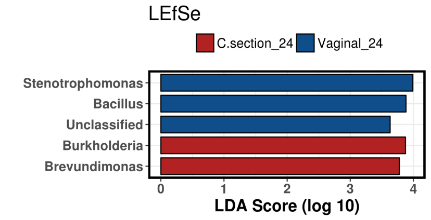


D

C

A

B

**Supplementary Figure S4.** LEfSe analysis determining discriminative taxa based on mode of delivery at week 1 (A), week 4 (B), week 8 (C) and week 24 (D)

**Supplementary Table S1.** Clinical Data of Participants

| **Subject Number** | **Subject Type** | **Gestation weeks** | **Birth Weight** | **Breast fed** | **Infant Sex** | **Race** | **Delivery Mode** | **Gravida** | **Para** | **BMI pre pregnancy** |
| --- | --- | --- | --- | --- | --- | --- | --- | --- | --- | --- |
| **PT1** | Full Term | 40 | 3200 | y | Female | Caucasian | VD | 1 | 1 | 22 |
| **PT2** | Full Term | 40 | 4460 | y | Male | Caucasian | VD | 3 | 2 | 23.2 |
| **PT3** | Full Term | 39 | 3460 | y | Female | Caucasian | LSCS | 5 | 3 | 26.3 |
| **PT4** | Full Term | 39 | 3180 | y | Female | Caucasian | LSCS | 1 | 1 | 22.4 |
| **PT5** | Full Term | 41 | 3880 | y | Male | Caucasian | VD | 1 | 1 | 28.7 |
| **PT6** | Full Term | 39 | 3230 | y | Male | Caucasian | LSCS | 3 | 3 | not noted |
| **PT7** | Full Term | 40 | 3850 | y | Male | Caucasian | VD | 1 | 1 | not noted |
| **PT8** | Full Term | 39 | 3200 | y | Female | Caucasian | VD | 2 | 2 | not noted |
| **PT9** | Full Term | 39 | 3460 | y | Female | Caucasian | VD | 3 | 3 | not noted |
| **PT10** | Full Term | 40 | 3400 | y | Male | Caucasian | VD | 4 | 3 | 21.27 |
| **PT11** | Full Term | 39 | 3420 | y | Male | Caucasian | LSCS | 2 | 2 | 25.64 |
| **PT12** | Full Term | 38 | 3100 | y | Male | Caucasian | VD | 2 | 2 | 20.2 |
| **PT13** | Full Term | 40 | 3450 | y | Female | Caucasian | VD | 1 | 1 | not noted |
| **PT14** | Full Term | 39 | 3120 | y | Male | Caucasian | VD | 1 | 1 | 21.23 |
| **PT15** | Full Term | 40 | 3700 | y | Female | Asian | VD | 2 | 2 | 26.44 |
| **PT16** | Full Term | 39 | 3240 | y | Female | Caucasian | VD | 6 | 4 | 37.58 |
| **PT17** | Full Term | 41 | 4600 | y | Male | Caucasian | VD | 2 | 1 | 28.56 |
| **PT18** | Full Term | 41 | 4450 | y | Female | Caucasian | VD | 2 | 2 | 23.5 |
| **PT19** | Full Term | 36 | 2840 | y | Male | Caucasian | VD | 2 | 2 | 25.83 |
| **PT20** | Full Term | 38 | 3440 | y | Male | African | VD | 3 | 2 | 42.46 |
| **PT21** | Full Term | 41 | 2970 | y | Female | Caucasian | VD | 1 | 1 | 24.33 |
| **PT22** | Full Term | 40 | 3900 | y | Female | Caucasian | VD | 2 | 2 | 21.6 |
| **PT23** | Full Term | 39 | 3430 | y | Male | Caucasian | VD | 1 | 1 | not noted |
| **PT24** | Full Term | 39 | 3760 | y | Female | Caucasian | VD | 1 | 1 | 27.11 |
| **PT25** | Full Term | 39 | 3780 | y | Male | Caucasian | VD | 2 | 1 | >40 |
| **PT26** | Full Term | 40 | 3320 | y | Male | Caucasian | VD | 2 | 2 | 22.4 |
| **PT27** | Full Term | 41 | 3260 | y | Male | Caucasian | VD | 4 | 3 | 23.05 |
| **PT28** | Full Term | 40 | 3890 | y | Female | Caucasian | VD | 2 | 2 | 22.21 |
| **PT29** | Full Term | 39 | 4740 | y | Female | Caucasian | LSCS | 1 | 1 | 23.46 |
| **PT30** | Full Term | 40 | 3280 | y | Female | Caucasian | VD | 1 | 1 | 28.03 |
| **PT31** | Full Term | 40 | 3850 | y | Female | Caucasian | VD | 3 | 3 | not noted |
| **PT32** | Full Term | 40 | 4080 | y | Female | Caucasian | VD | 3 | 3 | not noted |
| **PT33** | Full Term | 39 | 3560 | y | Male | Caucasian | LSCS | 2 | 2 | 24.98 |
| **PT34** | Full Term | 38 | 3360 | y | Male | Caucasian | LSCS | 4 | 4 | 29.35 |
| **PT35** | Full Term | 41 | 4620 | y | Male | Caucasian | VD | 2 | 2 | 28.43 |
| **PT36** | Full Term | 39 | 3800 | y | Female | Caucasian | LSCS | 1 | 1 | 37.95 |
| **PT37** | Full Term | 40 | 3930 | y | Male | Caucasian | VD | 1 | 1 | 24.11 |
| **PT38** | Full Term | 40 | 3470 | y | Female | African | VD | 1 | 1 | 22.37 |
| **PT39** | Full Term | 38 | 3620 | y | Male | Caucasian | VD | 2 | 3 | 32.12 |
| **PT40** | Full Term | 40 | 3680 | y | Female | Caucasian | VD | 4 | 3 | 81.41 |
| **PT41** | Full Term | 40 | 3160 | y | Female | Caucasian | VD | 1 | 1 | 23.6 |
| **PT42** | Full Term | 40 | 4100 | y | Male | Caucasian | VD | 2 | 2 | 23.62 |
| **PT43** | Full Term | 40 | 3910 | y | Male | Caucasian | VD | 3 | 3 | 24.53 |
| **PT44** | Full Term | 39 | 3140 | y | Male | Caucasian | LSCS | 3 | 3 | 28.2 |
| **PT45** | Full Term | 40 | 3760 | y | Male | Caucasian | VD | 2 | 2 | 30.48 |
| **PT46** | Full Term | 37 | 3250 | y | Male | Caucasian | LSCS | 1 | 1 | 28.22 |
| **PT47** | Full Term | 39 | 2970 | y | Male | Caucasian | VD | 2 | 2 | 26.19 |
| **PT48** | Full Term | 40 | 4670 | y | Male | Caucasian | LSCS | 1 | 1 | 32.1 |
| **PT49** | Full Term | 40 | 3620 | y | Male | Caucasian | VD | 2 | 2 | 24.99 |
| **PT50** | Full Term | 40 | 3670 | y | Male | Caucasian | VD | 1 | 1 | 21.96 |
| **PT51** | Full Term | 39 | 3930 | y | Male | Caucasian | VD | 5 | 3 | 26.76 |
| **PT52** | Full Term | 39 | 3050 | y | Female | Caucasian | LSCS | 2 | 2 | 28.91 |
| **PT53** | Full Term | 40 | 3300 | y | Female | Caucasian | VD | 3 | 3 | 21.98 |
| **PT54** | Full Term | 39 | 3920 | y | Female | Caucasian | LSCS | 2 | 2 | 29.98 |
| **PT55** | Full Term | 39 | 3560 | y | Male | Caucasian | VD | 3 | 2 | not noted |
| **PT56** | Full Term | 39 | 3490 | y | Female | Caucasian | VD | 3 | 2 | 26.53 |
| **PT57** | Full Term | 41 | 3920 | y | Male | Caucasian | VD | 3 | 3 | 20.42 |
| **PT58** | Full Term | 38 | 2990 | y | Female | Caucasian | LSCS | 3 | 2 | 27.47 |
| **PT59** | Full Term | 37 | 3020 | y | Female | Indian | VD | 2 | 1 | 27.53 |
| **PT60** | Full Term | 41 | 3660 | y | Male | Caucasian | LSCS | 1 | 1 | 22.55 |
| **PT61** | Full Term | 38 | 3240 | y | Male | Caucasian | VD | 5 | 1 | not noted |
| **PT62** | Full Term | 40 | 3670 | y | Female | Caucasian | LSCS | 2 | 2 | 22.55 |
| **PT63** | Full Term | 40 | 4190 | y | Female | Caucasian | VD | 2 | 2 | 23.4 |
| **PT64** | Full Term | 41 | 4670 | y | Male | Caucasian | VD | 4 | 4 | 24.89 |
| **PT65** | Full Term | 40 | 3500 | y | Female | Caucasian | VD | 1 | 1 | 25.6 |
| **PT66** | Full Term | 38 | 3480 | y | Male | Caucasian | VD | 4 | 2 | 24.24 |
| **PT67** | Full Term | 40 | 3500 | y | Female | Caucasian | VD | 1 | 1 | 20.37 |
| **PT68** | Full Term | 41 | 4150 | y | Female | Caucasian | VD | 1 | 1 | 22.55 |
| **PT69** | Full Term | 38 | 2920 | y | Male | Caucasian | VD | 2 | 2 | 19.84 |
| **PT70** | Full Term | 41 | 3700 | y | Female | Caucasian | VD | 2 | 1 | 25.93 |
| **PT71** | Full Term | 40 | 3780 | y | Male | Caucasian | VD | 3 | 3 | 22.84 |
| **PT72** | Full Term | 40 | 3980 | y | Male | Caucasian | VD | 5 | 1 | not noted |
| **PT73** | Full Term | 40 | 4680 | y | Male | Caucasian | VD | 3 | 2 | 28.14 |
| **PT74** | Full Term | 40 | 3960 | y | Male | Caucasian | VD | 3 | 2 | 28.37 |
| **PT75** | Full Term | 41 | 3750 | y | Female | Caucasian | VD | 1 | 1 | 22.02 |
| **PT76** | Full Term | 40 | 3380 | y | Female | mixed | VD | 1 | 1 | 23.15 |
| **PT77** | Full Term | not noted | not noted | y | Male | Caucasian | VD | 7 | 5 | 19.3 |
| **PT78** | Full Term | 39 | 3430 | y | Male | Caucasian | VD | 2 | 2 | 23.56 |
| **PT79** | Full Term | 39 | 3450 | y | Female | Caucasian | VD | 2 | 2 | 24.52 |
| **PT80** | Full Term | 40 | 4120 | y | Female | Caucasian | VD | 1 | 1 | 18.72 |
| **PT81** | Full Term | 41 | 3990 | y | Male | Caucasian | VD | 1 | 1 | 21.15 |
| **PT82** | Full Term | 38 | 3660 | y | Female | caucasian | LSCS | 3 | 2 | 21.4 |

***Supplementary Table S2.*** *Repeated measures statistical analysis at phylum level.*

| **Taxa** | **P-value** | **P.bonferroni** | **FDR** | **Week 4.coef** | **Week 8.coef** | **Week 24.coef** |
| --- | --- | --- | --- | --- | --- | --- |
| **Acidobacteria** | 9E-16 | 8.1E-15 | <0.001 | 2.3 | 0.011 | 0.2 |
| **Fusobacteria** | 1.9E-06 | 0.000015 | <0.001 | -0.28 | 0.48 | 2.1 |
| **Firmicutes** | 0.000015 | 0.0001 | <0.001 | -0.13 | 0.25 | 2.5 |
| **Bacteroidetes** | 0.000022 | 0.00013 | <0.001 | 0.91 | 0.25 | -1.5 |
| **Saccharibacteria** | 0.00038 | 0.0019 | <0.001 | 0.64 | 0.57 | 2.3 |
| **Proteobacteria** | 0.0042 | 0.017 | <0.01 | 1.7 | 1.1 | 0.58 |
| **Unclassified** | 0.024 | 0.072 | <0.05 | 0.48 | -0.86 | 0.75 |
| **Actinobacteria** | 0.035 | 0.072 | <0.05 | 0.82 | 0.15 | 1.2 |
| **Cyanobacteria** | 0.31 | 0.31 | 0.31 | 0.49 | 0.51 | -0.21 |
